# Baseline Distress and Effectiveness of Survivor Video Narratives on Cancer-Associated Distress in Botswana: A Pilot Study

**DOI:** 10.1200/GO-24-00474

**Published:** 2025-06-18

**Authors:** Yehoda M. Martei, Maanasa Gurram, Lebogang T. Mokokwe, Ngwao Ngwako, Keaobaka Kebuang, Dipho I. Setlhako, Peter Vuylsteke, Baaitse Bontswanetse, Tumisang Segadimo, Mosepele Mosepele, Lawrence N. Shulman, Frances Barg, Babe E. Gaolebale

**Affiliations:** ^1^ Department of Medicine (Hematology-Oncology Division), University of Pennsylvania, Philadelphia, PA; ^2^ University of Pennsylvania Perelman School of Medicine, Philadelphia, PA; ^3^ Botswana University of Pennsylvania Partnership, Gaborone, Botswana; ^4^ University of Botswana, Gaborone, Botswana; ^5^ Princess Marina Hospital, Gaborone, Botswana; ^6^ Department of Family Medicine and Community Health, University of Pennsylvania, Philadelphia, PA

## Abstract

**PURPOSE:**

To evaluate baseline distress among patients with breast cancer in Botswana, and assess the impact of culturally tailored peer survivor video narratives on distress and its mediators.

**METHODS:**

We enrolled patients with stage I-IV breast cancer at Princess Marina Hospital. A Setswana-translated National Comprehensive Cancer Network distress thermometer (DT) and problem list (PL) were used for distress screening. DT score of ≥4 was considered a positive screen for moderate to high (moderate-high) distress. We analyzed independent PL factors associated with moderate-high distress using logistic regression. Participants then watched one to two videos and completed a postintervention DT/PL assessment after each video at 4 and 8 weeks. We conducted descriptive statistics to explore the impact of the videos.

**RESULTS:**

One hundred six participants were enrolled, of whom 103 completed baseline DT and 106 completed baseline PL. Sixty-seven percent (69/103) of participants screened positive for moderate-high distress at baseline. Fear (odds ratio [OR], 11.25 [95% CI, 1.66 to 76.49]; *P* = .01) and appearance (OR, 4.96 [95% CI, 1.03 to 23.80]; *P* = .046) were PL factors significantly associated with moderate-high distress in the multivariable model. Sixty-eight and 47 participants completed postvideo assessments at approximately 4 and approximately 8 weeks, respectively. The greatest impact was observed at 8 weeks after watching two videos—29.8% of participants with moderate-high distress had no or mild distress. Similarly, there was a 29% (44%-15%; *P* = .005) and 17% (32%-15%; *P* = .03) absolute decrease from baseline to 8 weeks, in the proportion of patients who identified fear and appearance as sources of distress, respectively.

**CONCLUSION:**

Two thirds of patients with breast cancer screened positive for moderate-high distress. Fear and appearance were sources of distress significantly associated with a positive screen. Our results show promising potential of peer survivor videos to mitigate distress and its potential mediators among patients with breast cancer.

## INTRODUCTION

Individuals with a new diagnosis of breast cancer and those undergoing cancer-specific treatment may experience distress related to direct physical effects of the cancer, treatment-related adverse effects, psychosocial stressors, such as financial toxicity, fear of recurrence and death, and other associated distress factors.^1,2^ Distress is prevalent among patients with cancer, and is associated with worse outcomes.^3,4^ On average, 20%-40% of patients with cancer experience clinically significant levels of distress.^5^ Among patients with cancer, moderate to high (moderate-high) levels of distress have been associated with ineffective coping abilities, treatment deviations from standard of care, prolonged hospitalizations, lower quality of life, increased risk of suicidal ideation, and worse mortality.^6-8^ Furthermore, among patients with breast cancer, psychosocial distress is associated with decreased adherence to curative-intent endocrine therapy,^9^ and an increased risk of all-cause mortality (adjusted hazard ratio, 1.46 [95% CI, 1.02 to 2.09]).^10^

**CONTEXT**

**Key Objective**
What is the level of distress among patients with breast cancer in Botswana. Can narratives from other breast cancer survivors from Botswana potentially mitigate cancer-related distress in this population?
**Knowledge Generated**
Approximately two thirds of participants enrolled in this study met criteria for moderate-high distress. Among study participants, fear and appearance were significantly associated with distress. Watching two survivor narrative videos from other Botswana breast cancer survivors decreased distress, fear, and appearance at 8 weeks.
**Relevance**
The high rate of cancer-associated distress among breast cancer survivors underscores the need for routine distress screening. Survivor stories are potentially impactful in minimizing distress in this breast cancer population in Botswana.

Subsequently, interventions addressing distress are important for individuals diagnosed with breast cancer and other cancers. Among patients with other chronic diseases, interventions that address disease-associated distress have been shown to improve quality-of-life outcomes and are cost-effective.^11-13^ In 2012, the American College of Surgeons Commission on Cancer instituted a mandate requiring all member institutions to implement routine distress screening and referral, for cancer center accreditation.^14^ Similarly, the National Comprehensive Cancer Network (NCCN) and ASCO have made similar recommendations for distress screening of all patients attending cancer clinics in the United States.^15^ The NCCN distress thermometer (DT), which captures distress levels and associated problems in a problem list (PL), is a common screening tool.^16^ In a 2018 survey of NCCN member institutions, 87% of surveyed institutions reported currently conducting routine screening for distress.^17^

Similar data on the routine assessment of clinical distress and referral for supportive services for patients with breast cancer and other cancers are lacking in low- and middle-income countries (LMICs). A recent study conducted in India showed that 53.5% of patients with cancer had moderate-high levels of clinical distress,^18^ and a study in Ethiopia also showed higher levels of distress among patients with cancer compared with European counterparts.^7^ These few publications suggest that distress levels among patients with cancer in LMICs may be higher than in high-income countries. There is therefore a critical need for routine assessment of distress levels and linkage to appropriate care. The aims of this pilot study conducted in Botswana (Southern Africa) were to (1) evaluate baseline distress levels among patients with breast cancer undergoing routine care using the NCCN DT and analyze the PL factors associated with moderate-high distress levels; and (2) evaluate the effectiveness of peer survivor narrative videos on moderate-high distress and its potential mediators.

## METHODS

### Study Design and Participants

This was a single-arm pilot study that prospectively enrolled patients attending the breast cancer multidisciplinary clinic or oncology clinic at Princess Marina Hospital with a confirmed diagnosis of breast cancer. We used a convenience sampling approach. The inclusion criteria were patients age 18 years and older, with pathologically confirmed diagnosis of stage I-IV breast cancer at any point in their cancer treatment. Participants were enrolled between October 2021 and June 2022. The study was initially limited to stage I-III breast cancer but later extended to all eligible patients with breast cancer regardless of stage to increase generalizability of our results and increase enrollment. Informed consent was obtained before study enrollment.

### NCCN DT

The NCCN DT was first developed as a tool for screening distress in patients with cancer, subsequently a PL was added to the DT to identify sources of distress, to provide linkage to resources to address the respective PL items.^19,20^ The DT is a visual analog scale representing patient-reported distress on a scale of 0 (no distress) to 10 (extreme distress). The PL is categorized under the following domains: practical, physical, emotional, familial, and spiritual/religious, representing a biopsychosocial-spiritual model of distress in cancer.^21^ The NCCN DT has been validated and translated to more than 60 languages.^22^ It was recently validated for distress assessment in a South African population.^23^ For the purposes of this study, version 2.2020 was translated to Setswana, through iterative discussions with local experts on the research team to ensure cultural sensitivity and preservation of the intended meanings of the DT and PL. The Setswana-translated NCCN distress survey was pilot-tested among three patients, and the final version was administered by a trained research nurse and a medical student at the University of Botswana.

### Study Procedures

The development of the survivor video narrative intervention by Botswana breast cancer survivors has been previously published by Martei et al.^24^ Each video was approximately 15 minutes long and focused on different breast cancer survivors from Botswana telling their story in a structured interview format. The content of the videos addressed breast cancer stigma, fears, myths, treatment, survivorship, and other topics informed by previous qualitative research on barriers to breast cancer treatment adherence.^24^ Three completed videos from three different survivors were used in this pilot study, all varying in content on the basis of the individual survivor's experiences with the common goal of promoting education and treatment adherence. All consenting participants provided information on clinical and demographic characteristics at baseline, as well as a baseline DT and PL assessment. Participants then watched one of the three video options on a tablet in-person and completed video 1 DT/PL post-assessment at approximately 4 weeks or when their next clinic follow-up was scheduled. Participants who returned for an additional in-person follow-up visit were asked to watch another video (video 2), thereby completing two videos in total. Those who watched video 2 completed another DT/PL post-assessment at approximately 8 weeks. Repeating DT/PL assessments is aligned with NCCN recommendations to assess distress at interval visits.^15^

All study personnel and patients adhered to Botswana's strict COVID-19 precautions, and the country's high vaccination rate allowed for safe in-person administration of the DT/PL assessments. All participants were provided information for provider and peer cancer survivor support services through the Cancer Association of Botswana.

### Statistical Analyses

DT score was assessed as a continuous outcome. The data distribution for DT scores was tested using skewness/kurtosis test for normality, with probability for skewness and kurtosis >0.05. Subsequently parametric testing was used to compare the means of DT scores for the various demographic and clinical groups. The DT score was also assessed as a binary outcome, with DT score of ≥4 representing a positive screen for moderate-high levels of distress and <4 representing no or mild levels of distress.^25^ Univariate and multivariate logistic regression analyses were used to explore PL items that were associated with a positive screen for moderate-high distress. The covariates included in the model were PL factors included in the NCCN 2.2020 version. Covariates associated with moderate-high distress in the univariate analyses with *P* value of <.1 were included in the multivariate logistic regression model to identify independent sources of distress associated with a positive screen for moderate-high distress. We tested for multicollinearity of the variables in the multivariable logistic regression model using variance inflation factor (VIF). VIF <10 indicates no collinearity between the variables.

We evaluated the impact of the survivor video narratives by describing the proportion of patients with moderate-high distress at baseline who transitioned to no or low distress (DT score <4) after video 1 (video 1 post-assessment at approximately 4 weeks), and those who made this transition after video 2 (video 2 post-assessment at approximately 8 weeks). Focusing on only PL items independently associated with moderate-high distress as potential mediators of moderate-high distress, we explored the impact of the survivor video narratives on the respective PL items by comparing the proportions endorsing the problem at baseline and in the video 1 post-assessment (at 4 weeks), as well as baseline compared with video 2 post-assessment (at 8 weeks). All tests were conducted using two-sided *P* < .05 to conclude statistical significance. All statistical analyses were conducted using STATA 14.0.

### Ethical Clearance

This pilot study was approved by the institutional review boards at the University of Pennsylvania (842894), the University of Botswana Institutional Review Board (Ref: UBR/RES/IRB/BIO/184), the Botswana Human Research Development Committee (HPDME13/18/1), the Ministry of Health, and Princess Marina Hospital (PMH 2/11AII).

## RESULTS

### Patient Characteristics

**FIG 1:**
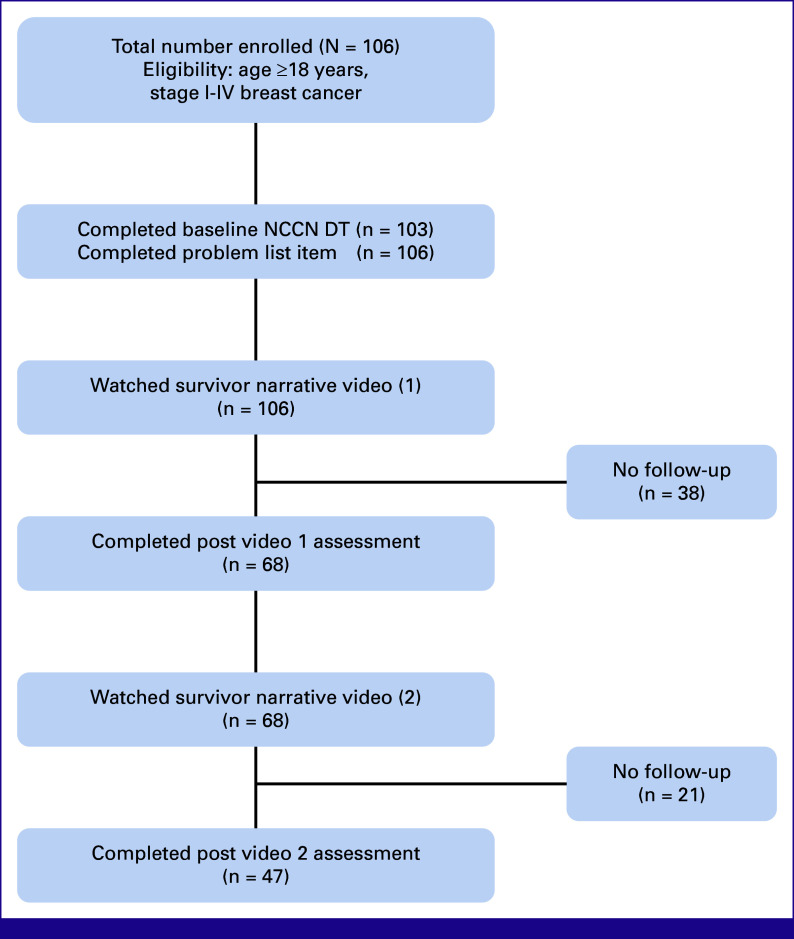
Patient flow diagram showing the number of participants enrolled at baseline and the number of participants who completed interval DT and PL assessments after the video interventions. DT, distress thermometer; NCCN, National Comprehensive Cancer Network; PL, problem list.

A total of 106 patients with breast cancer, all women, with stage I-IV disease were enrolled in this study from October 2021 to June 2022. Of these, 103 patients completed baseline DT, 106 completed baseline PL, 68 completed the post-assessment after watching the first video at approximately 4 weeks, and 47 completed the post-assessment after the second video at approximately 8 weeks. Figure 1 displays the flow diagram of patients enrolled at baseline and those who completed follow-up assessments. More than half of the 103 patients who completed baseline DT were younger than 50 years, and 34% of the patients with breast cancer enrolled were those living with HIV. Table 1 summarizes additional patient demographic and clinical characteristics with corresponding mean DT scores and 95% CI.

**TABLE 1:** Summary of Patient Clinical and Demographic Characteristics, and Mean Distress Scores Across the Respective Groups

Number of Patients (sample size)	103	Percentage (%)	Mean DT Score	95% CI (lower bound)	95% CI (upper bound)
Age, years					
<50	53	51.5	4.79	3.99	5.60
≥50	50	48.5	4.02	3.29	4.74
Stage					
Stage I	3	2.9	2.33	0	6.97
Stage II	28	27.2	4.50	3.43	5.57
Stage III	55	53.4	4.40	3.69	5.11
Stage IV	9	8.7	4.90	2.66	7.12
Missing	8	7.8			
HIV status					
Positive	35	34.0	4.57	3.78	5.37
Negative	68	66.0	4.34	3.62	5.06
Highest education level attained					
Tertiary	23	22.3	5.0	3.70	6.30
Senior secondary	17	16.5	4.10	2.67	5.46
Junior secondary	29	28.2	5.0	3.90	6.10
Primary	26	25.2	3.81	2.93	4.68
None	8	7.8	3.38	1.98	4.77
Location^a^					
Urban	28	27.2	4.90	3.80	6.10
Rural	75	72.8	4.20	3.60	4.80
Marital status					
Married/partnered	36	35.0	3.97	3.00	4.94
Not married/widowed	67	65.0	4.66	4.00	5.31
Employment status					
Employed	47	45.6	4.70	3.90	5.51
Not employed	56	54.4	4.18	3.44	4.92
Income level					
≤BWP 600	47	45.6	4.30	3.50	5.10
BWP 601-10,000	45	43.7	4.70	3.80	5.60
BWP ≥10,000	11	10.7	3.80	2.30	5.40

Abbreviations: BWP, Botswana Pula (Botswana's currency); DT, distress thermometer.

^a^ Urban represents Gaborone and surrounding areas, while rural represents outside Gaborone.

### Assessment of Moderate-High Distress and Association With PL Items

Of the 103 patients with baseline DT information, 69 (67%) screened positive for moderate-high distress at baseline (DT scores of ≥4). Patients with stage I disease, with lower levels of education (ie, none or primary levels only), and patients who were married or had partners had lower mean baseline distress scores <4. Additionally, patients with monthly incomes ≥Botswana Pula 10,000 (US $1,000 equivalent) had a mean DT score <4. All other demographic and clinical groups had mean DT scores ≥4 (Table 1).

**FIG 2:**
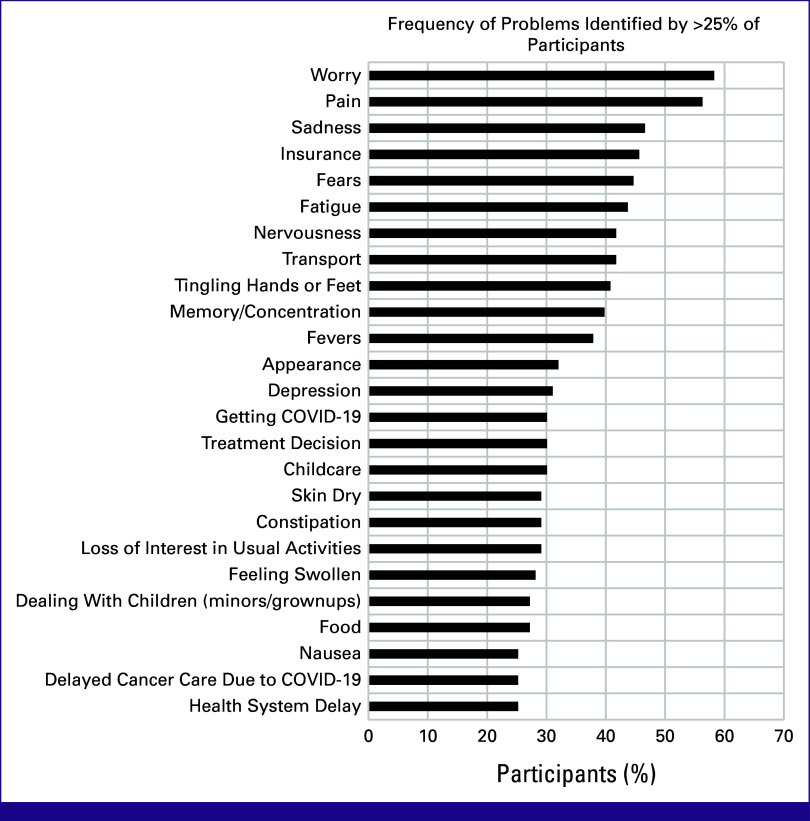
Frequency of problems identified by >25% of participants enrolled.

There was a wide distribution of PL items identified across all the domains. Because of the overlap of the COVID-19 pandemic and the study period, there were also common concerns related to the COVID-19 pandemic. The most commonly identified PL items were in the emotional domain (Data Supplement, Table S1). Of the 106 patients with baseline PL completed, 60 (56.6%) endorsed worry and 58 (54.7%) endorsed pain. Figure 2 displays the percentage of patients endorsing specific PL items for problems identified by more than 25% of patients.

Table 2 summarizes the results of the univariate and multivariate logistic regression analyses showing the PL factors associated with screening positive for moderate-high distress. In the univariate analysis, the following PL items were significantly associated with screening positive for moderate-high distress at *P* < .05: work/school, childcare, food, dealing with children (minors/grownups), fears, nervousness, sadness, worry, appearance, eating, getting around, and nausea. VIF for all covariates was <10 indicative of low likelihood of collinearity between variables, subsequently all variables meeting criteria in the univariate model were included in the multivariable model. All PL items with significance of <0.1 were included in the final multivariable model. In the full model, appearance (odds ratio [OR], 4.96 [95% CI, 1.03 to 23.80]; *P* = .046) and fear (OR, 11.25 [95% CI, 1.66 to 76.49]; *P* = .01) were significantly associated with screening positive for moderate-high distress in this population after controlling for other PL items (Table 2).

**TABLE 2:** Univariate and Multivariate Logistic Regression Analyses of PL Items Associated With High Distress, DT ≥4

PL	Univariate Analysis		Multivariable Analysis	
	OR (95% CI)	*P*	OR (95% CI)	*P*
Insurance	2.28 (0.97 to 5.39)	.06	1.02 (0.26 to 4.00)	.98
Transport	2.2 (0.92 to 5.28)	.07	1.39 (.38 to 5.03)	.62
Work/school	16.6 (2.12 to 128.35)	<.001	12.85 (0.85 to 192.95)	.07
Childcare	3.5 (1.21 to 10.19)	.01	0.36 (0.04 to 2.84)	.33
Food	4 (1.26 to 12.70)	.009	0.84 (0.10 to 6.86)	.87
Dealing with children (minors/grownups)	4.52 (1.24 to 16.44)	.009	6.0 (0.79 to 45.65)	.08
Depression	2.19 (0.83 to 5.75)	.10	0.35 (.05 to 2.28)	.28
Fears	6.44 (2.36 to 17.55)	<.001	11.25 (1.66 to 76.49)	.01
Nervousness	3.34 (1.33 to 8.41)	.007	0.33 (0.05 to 2.19)	.25
Sadness	4.48 (1.78 to 11.31)	<.001	0.71 (0.11 to 4.48)	.72
Worry	5.12 (2.11 -12.44)	<.001	2.98 (0.51 to 17.42)	.23
Loss of interest in usual activities	2.5 (0.91 to 6.84)	.06	0.94 (0.14 to 6.51)	.95
Appearance	5.44 (1.75 to 17.13)	.001	4.96 (1.03 to 23.81)	.046
Eating	3.06 (0.95 to 9.82)	.04	1.38 (0.26 to 7.31)	.71
Getting around	6.95 (0.86 to 55.86)	.02	5.08 (0.42 to 61.42)	.2
Nausea	5.17 (1.43 to 18.70)	.004	2.83 (0.55 to 14.51)	.21
Nose dry/congested	3.71 (0.79 to 17.51)	.06	0.52 (0.06 to 4.59)	.56
Housing	—	—		
Treatment decision	2.06 (0.78 to 5.41)	.13		
Health system delay	1.47 (0.55 to 3.92)	.44		
Dealing with partner	1.52 (0.29 to 7.98)	.61		
Ability to have children	3.73 (0.44 to 31.59)	.17		
Family health issues	2.4 (0.63 to 9.07)	.17		
Spiritual or religious concerns	0.98 (0.17 to 5.66)	.99		
Traditional healing concerns	—	—		
Catching the COVID-19 virus	0.85 (0.35 to 2.07)	.73		
Losing a loved one to COVID-19	2.26 (0.69-7.40)	.15		
Delayed cancer care due to COVID-19	1.15 (0.44 to 2.99)	.78		
Bathing	1.52 (0.29 to 7.98)	.61		
Breathing	2.7 (0.56 to 13.14)	.18		
Changes in urination	1.22 (0.39 to 3.80)	.73		
Constipation	1.52 (0.59 to 3.90)	.37		
Diarrhea	2.1 (0.42 to 10.47)	.34		
Fatigue	0.82 (0.36 to 1.86)	.63		
Feeling swollen	1.42 (0.55 to 3.65)	.46		
Fevers	1.18 (0.50 to 2.77)	.71		
Indigestion	2.4 (0.63 to 9.07)	.17		
Memory/concentration	1.32 (0.57 to 3.11)	.51		
Mouth sores	3.73 (0.44 to 31.59)	.17		
Pain	1.22 (0.54 to 2.80)	.63		
Sexual	0.45 (0.12 to 1.69)	.24		
Skin dry	1.52 (0.59 -3.90)	.37		
Sleep	1.53 (0.54 to 4.31)	.42		
Substance abuse	0.64 (0.13 to 3.02)	.57		
Tingling hands or feet	0.81 (0.35 to 1.87)	.63		

Abbreviations: DT, distress thermometer; OR, odds ratio; PL, problem list.

Of the 68 patients who completed the post-assessment after video 1 at 4 weeks, 42.6% experienced a reduction in DT score compared with baseline and 28.8% of patients who screened positive for moderate-high distress at baseline had no or mild distress after watching video 1. Forty-seven patients watched both video 1 and video 2 and completed the post-assessment after video 2 at approximately 8 weeks. Of those, 48.9% had a reduced DT score compared with baseline DT score at enrollment and 29.8% of patients who experienced moderate-high distress at baseline had no or mild distress after watching a total of two videos.

**FIG 3:**
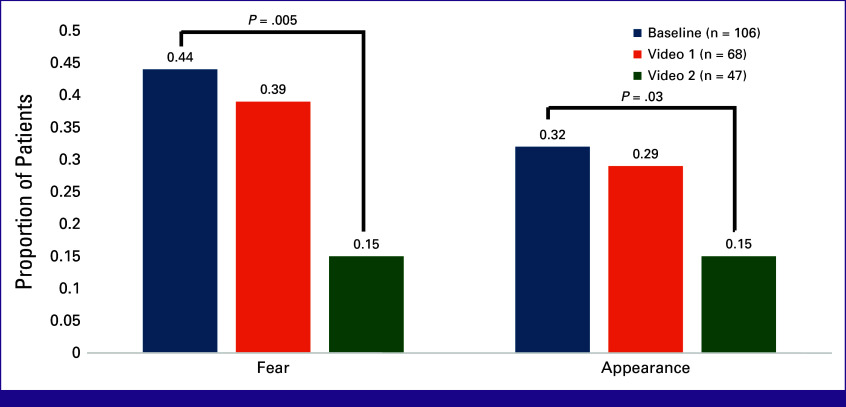
Impact of survivor video narratives on proportion of patients who selected fear and appearance as sources of distress at baseline, and at 4 and 8 weeks (after video intervention).

Given that fear and appearance were significantly associated with screening positive for moderate-high distress, we explored the effect of the survivor narrative videos on these PL items. Figure 3 displays the respective proportions of patients who endorsed fear and appearance as PL items at baseline, in the post-assessment for video 1 (at approximately 4 weeks), and in the post-assessment for video 2 (at approximately 8 weeks). In exploratory analysis, the largest impact of the videos was observed after two videos. At approximately 8 weeks, of the 47 patients who completed post-assessment video 2, there was a 29% (44%-15%; *P* = .005) absolute decrease in the percent of patients identifying fear as a source of distress and a 17% (32%-15%; *P* = .028) decrease in the percent of patients identifying appearance as a source of distress compared with the respective proportions at baseline (Fig 3).

## DISCUSSION

This study using the NCCN DT/PL to screen for distress and possible sources of distress in a population of patients with breast cancer in Botswana enrolled 106 patients, with an overwhelming majority of patients screening positive for moderate-high levels of distress. We also identified significant sources of distress in the univariate and multivariate logistic regression analyses. Importantly, fear and appearance were significantly associated with a positive screen, with patients with either of these concerns having elevated odds of screening positive for moderate-high distress. Furthermore, stratifying by clinical and demographic variables showed that patients with stage I disease, higher incomes, and those who were married or partnered had mean DT scores <4. Additionally, patients with lower educational levels had lower distress scores. However, across most strata, mean DT scores were in the range of moderate-high levels of distress, emphasizing the high prevalence of distress in this group of patients with breast cancer. Finally, the culturally tailored peer survivor video narratives reduced distress among those who screened positive for moderate-high distress and significantly reduced the proportion of patients reporting fear and appearance as sources of distress.

This is one of the very few studies examining the use of the NCCN DT/PL as a rapid screening tool for distress among patients with breast cancer in Africa. A recent study validated a version of the NCCN DT to screen for distress and patient-reported symptoms in a South African population.^23^ The results of our study suggest that the lack of routine distress screening means that a substantial proportion of patients with breast cancer with distress may be underdiagnosed and undertreated for psychosocial distress. The proportion of patients screening positive for moderate-high distress are similar to proportions reported among patients with cancer in other LMICs. A recent study from India showed that majority of adult patients with cancer were distressed.^18^ Another study in Egypt using a modified NCCN DT to screen patients with COVID-19 in Egypt showed that 60.4% of patients experienced distress, similar to our finding of 67% of patients experiencing moderate-high distress.^26^ The study from Egypt also reported similar demographic factors significantly associated with distress, including age and marital status. In high-income countries, numerous publications in the United States and similar countries have shown a wide range of proportions of patients with moderate-high distress levels, which vary by cancer type, treatments received, and predictors of distress similar to those found in the LMICs, such as age, sex, and marital status.^5,27,28^

Similar to previous studies, emotional factors such as fear, for example, fear of disease progression and recurrence, were predictors of moderate-high levels of distress.^29,30^ Interestingly, we also identified appearance as an independent factor associated with a positive screen. In this sociocultural context, previous studies have suggested that fear and appearance could be related to important stigma experiences faced by patients with breast cancer. For instance, studies conducted in several countries within Africa have identified fear of mastectomy, fear of being stigmatized, fear of social exclusion, and fear of financial toxicity as perceived stigma experiences among patients with breast cancer.^31-34^ Additionally, appearance may be related to the perception of disfigurement and disability associated with undergoing a mastectomy for breast cancer, as well as stigmata of cancer treatment side effects, such as alopecia, and nail and skin discoloration, which are important stigma markings in this context.^32,35^

The results of our pilot intervention using a peer-led breast cancer survivor video narratives on the basis of behavioral change theory and behavioral change techniques is promising in terms of its impact on distress and its potential mediators. In low-resource settings, despite the large proportion of patients with moderate-severe levels of distress, only 8.6% received referral to support services.^18^ Barriers identified for referral to psychosocial services include inadequate counseling staff, lack of awareness among providers, time, and financial constraints.^17^ The peer survivor video narratives is a feasible and potentially effective intervention that could be leveraged to address cancer-associated distress and potential mediators of distress. Peer narratives is an evidence-based intervention that has been used in other contexts to promote positive health behaviors such as cervical cancer screening and mammography uptake.^24,36-39^

Our study has several limitations. As this was a single-arm pilot study intended to assess feasibility and provide preliminary data to support a larger hybrid efficacy and implementation trial, there was no control arm, and the sample size of the study was small, therefore we could have missed significant findings in subgroups of patients. However, proportions of patients who screened positive for moderate-high distress were similarly high to those reported in the literature from LMICs. Furthermore, there were similarities in the association between the sources of distress in the emotional domain and moderate-high distress. There was also a high attrition rate at 4 weeks and 8 weeks, which may have affected the results. It is also possible that the reductions in stress were the result of time and adjustment to having a diagnosis of breast cancer, rather than to the video interventions. The high attrition rate may be due to the fact that patients were not reimbursed to return for additional visits, so their ability to complete video 2 was based on alignment with their appointment schedule. It is also possible that patients who did not benefit from the video the first time were less likely to return and also contributed to attrition rates. To truly understand the role of video intervention, a randomized study with high retention must be conducted. It is possible that COVID-19 may have exacerbated general distress and contributed to higher patient-reported distress level. However, although COVID-19–associated concerns were prevalent, our univariate did not show a significant association between COVID-19–related concerns and distress. Finally, there is a potential bias from the oncologist providing more education and support for patients with higher distress. Although we did not set out to purposively blind providers, data were collected in Research Electronic Data Capture, so not directly available to providers. This bias is therefore less likely; however, patients may be more empowered after completing the NCCN DT/PL and videos to ask for more support during their visit.

Despite these limitations, this study is important for increasing awareness of the elevated levels of distress among patients with breast cancer seeking care at a tertiary hospital in Botswana. The high levels of distress reported warrant the need to introduce routine distress screening as part of patient care even in low-resource settings. Analyzing baseline and interval measurements of DT/PL allowed us to assess the impact of survivor video narratives on changes in distress and sources of distress over time. Our analysis identified potential mediators of distress, which could guide tailored approaches to the management of distress and associated stigma experiences in these settings. Further studies are needed to assess implementation, effectiveness, and sustainability of culturally tailored peer survival video narratives as a part of a bundle intervention to address cancer-associated distress.
